# Patterns of Psychiatric Outpatient Practice in Taiwan: A Nationwide Survey

**DOI:** 10.3390/ijerph13100955

**Published:** 2016-09-28

**Authors:** Ying-Xiu Dai, Mu-Hong Chen, Tzeng-Ji Chen, Ming-Hwai Lin

**Affiliations:** 1 Department of Family Medicine, Taipei Veterans General Hospital, No. 201, Sec. 2, Shi-Pai Road, Taipei 112, Taiwan; daiinxiu@gmail.com (Y.-X.D.); tjchen@vghtpe.gov.tw (T.-J.C.); 2 School of Medicine, National Yang-Ming University, No. 155, Sec. 2, Linong Street, Taipei 112, Taiwan; kremer7119@gmail.com; 3 Department of Psychiatry, Taipei Veterans General Hospital, No. 201, Sec. 2, Shi-Pai Road, Taipei 112, Taiwan

**Keywords:** national health programs, physician’s practice patterns, psychiatry, Taiwan

## Abstract

(1) Background: Limited studies have utilized nationwide data to assess the patterns of psychiatric practice in other countries. In this study, data from the National Health Insurance Research Database in Taiwan (NHIRD-TW) for 2012 was analyzed to determine the patterns of psychiatric outpatient practice in Taiwan; (2) Methods: To determine the patterns of psychiatric outpatient practice in Taiwan, the data were drawn from the datasets of Taiwan’s National Health Insurance Research Database for 2012, with 619,760 records of outpatient visits representing 1/500 of all the claims in Taiwan for that year. The analysis of psychiatric outpatient visits included patient demographics, diagnoses, and prescribed medications; (3) Results: Neurotic disorders were the most prevalent diagnoses (43.1%, n = 5714). Hypnotics-sedatives and anxiolytics were prescribed in 51.7% (n = 6850) and 39.1% (n = 5181) of psychiatric visits, respectively, with zolpidem being the most commonly prescribed drug (22.6%, n = 2998); and (4) Conclusion: Hypnotics and sedatives were widely prescribed for the outpatient population, and zolpidem had the highest annual prevalence of use. These findings deserve the attention of clinicians and policy makers for monitoring the abuse and dependence of these agents and subsequent adverse events.

## 1. Introduction

With ongoing innovations in psychotropic medications, advances in rehabilitation models, and reforms to health care insurance systems, psychiatric practice has changed substantially in recent years. Comprehensive and detailed information is necessary in order to better understand and improve upon psychiatric services. Previous pharmacoepidemiological studies have analyzed the use of psychotropic drugs in various nationwide populations [1,2,3]. Many studies have also investigated the epidemiology of psychiatric visits to emergency departments [4,5,6,7]. Some reports have reviewed the trends and patterns of office-based psychiatric care in the United States based on the National Ambulatory Medical Care Survey, the National Hospital Ambulatory Medical Care Survey, and the National Health and Nutrition Examination Survey [8,9,10]. However, studies utilizing nationwide data to assess the patterns of psychiatric practice in other countries are limited. The Taiwan National Health Insurance (NHI) program was established in 1995 to provide health care to all residents in Taiwan. Currently, 99.9% of Taiwan’s population is covered by the NHI program. It is a government-administered insurance-based national healthcare system. Taiwan has a single payer system for healthcare. The characteristics of the NHI system include good accessibility, comprehensive population coverage, short waiting times, relatively low costs, and a national health insurance databank for planning, monitoring, and evaluating health services. However, the gatekeeper role of family doctors is weak in Taiwan. Taiwanese people are able to choose their healthcare providers freely, including any specialty and level of medical service. The National Health Insurance Administration of the Ministry of Health and Welfare has released all de-identified claims data dating back to 1999 for academic research. The National Health Insurance Research Database in Taiwan (NHIRD-TW) contains the original medical claims data submitted by all service providers in the nation. In this study, data from the NHIRD-TW for 2012 was analyzed to determine the patterns of psychiatric outpatient practice in Taiwan.

## 2. Materials and Methods

### 2.1. Data Collection

We assessed the files sampled from those of the year 2012 (S_CD20120.DAT and S_OO20120.DAT of NHIRD). The dataset “CD” means collection of all the outpatient visit files; on the other hand, the dataset “OO” refers to the outpatient order files. These two collections of files—containing a total of 619,760 medical records—were acquired by a 0.2% sampling ratio from the CD and OO datasets for 2012, excluding dentistry and traditional Chinese medicine. In this study, the data of outpatient visits encompassed visits to psychiatrists in outpatient departments of general hospitals, mental health hospitals, and physician clinics. It also included services from daycare wards and community rehabilitation centers, while the data of inpatient claims were not included in the study. All the diagnosis codes in every medical record were analyzed and demonstrated in the distribution of diagnoses among psychiatric outpatients, including psychiatric and nonpsychiatric diagnoses (Table 1).

### 2.2. Study Population

Analysis was conducted on the datasets of outpatient visit files and outpatient order files sampled from the NHIRD-TW for 2012. The sampled dataset contained 13,261 records, representing 1/500 of all the claims for outpatient services in 2012. Disease categories based on the International Classification of Diseases, 9th Revision, Clinical Modification (ICD-9-CM) classification were used to analyze the diagnoses in the outpatient visits. The Anatomical Therapeutic Chemical (ATC) classification system was used to assess prescription patterns. Outpatient visits were stratified by patient gender, age, diagnoses, and medications. The patterns of nonpharmacological treatments were also examined.

### 2.3. Statistical Analysis

The data were analyzed using the programming software Perl version 5.20.2 (Perl, Walnut, CA, USA) for data processing and using the statistical software SPSS version 22.0 (IBM, Armonk, NY, USA) for statistical analysis. Pearson’s χ^2^ test was used for group comparisons. A *p*-value < 0.05 (two-tailed) was considered statistically significant. The study was approved by institutional review board of Taipei Veterans General Hospital according to the Republic of China law (VGHIRB No.: 2013-10-001CE).

## 3. Results

### 3.1. The Demographics of Psychiatric Outpatients

**Figure 1:**
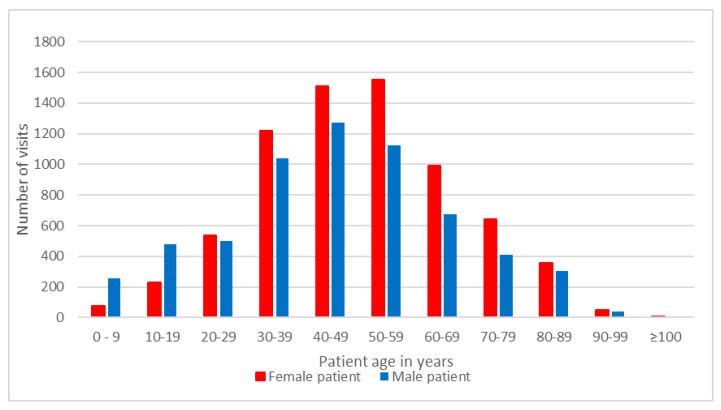
Number of outpatients visits by patient age and gender.

We identified 13,261 psychiatric outpatient visits, which accounted for 2.1% of all ambulatory care visits (n = 619,760) in 2012. Of the psychiatric outpatient visits, 7159 visits (54.0%) were made by female patients, and 6203 visits (46.0%) were made by male patients. In terms of age, patients aged 30–39 years (17.0%), 40–49 years (21.0%), and 50–59 years (20.2%) comprised the majority of the outpatients in 2012 (Figure 1). A predominance of male patients in the psychiatric outpatient visits was found among patients aged 0–9 and 10–19, with male-to-female ratios of 3.47 and 2.07, respectively, while male-to-female ratios ranging between 0.73 and 0.93 were observed in patients aged 20 and older.

### 3.2. Distribution of Outpatient Visits by Patient’s Diagnosis

Table 1 shows the distribution of diagnoses among psychiatric outpatients with all diagnoses at each claim taken into account. Diagnoses of mental disorders were found for 98.0% (n = 12,998) of psychiatric outpatient visits. In terms of the first diagnosis code listed in the medical claims, neurotic disorders (n = 4281, 32.3%), affective disorders (n = 2854, 21.5%), and schizophrenic disorders (n = 2241, 16.9%) were the major diagnoses at all facility levels. Among visits to physician clinics (n = 4379), 50.2% (n = 2198) of the first diagnosis codes were neurotic disorders. Neurotic disorders (ICD-9-CM diagnosis code 300) correspond to dysthymic disorder, anxiety disorders, obsessive-compulsive disorders, somatoform disorders, dissociative disorders, and factitious disorders in the Diagnostic and Statistical Manual of Mental Disorders (DSM) classification system. Considering the diagnostic distribution in terms of patient gender, there were more female patients (n = 2594, 60.6%) than male patients (n = 1687, 39.4%) among the patients with neurotic disorders (n = 4281). Among the patients with affective disorders (n = 2854), male patients accounted for 37.3% (n = 1064) and female patients accounted for 62.7% (n = 1790) of outpatient visits. Among the patients with schizophrenic disorders (n = 2241), 53.4% (n = 1196) of the visits were made by male patients, while 46.6% (n = 1045) were made by female patients. Constipation, hypertension, and diabetes mellitus were the most common medical diagnoses among the psychiatric outpatients. In patients with constipation, affective disorders (29.3%) were the most prevalent diagnoses, followed by schizophrenic disorders (28.8%) and neurotic disorders (23.7%). Among patients with diabetes mellitus, schizophrenic disorders (38.8%) were far more prevalent than affective disorders (20.1%) and neurotic disorders (10.5%).

### 3.3. Distribution of Outpatient Visits by Pharmacological and Nonpharmacological Treatments

As shown in Table 2, among nonpharmacological treatments, supportive psychotherapy was the most commonly applied treatment, followed by individual re-educative psychotherapy. Other infrequently performed treatments included behavioral therapy, supportive group psychotherapy, re-educative group psychotherapy, activity therapy, biofeedback therapy, and occupational therapy. With regard to patient gender, supportive psychotherapy was applied to 983 (46.4%) male patients and 1134 (53.6%) female patients. More female patients (n = 937, 59.4%) than male patients (n = 641, 40.6%) received re-educative group psychotherapy.

In terms of pharmacological treatments, 92.6% (n = 12,279) of outpatients were managed with medications, and 56.3% (n = 7475) of outpatients received three or more medications. Among visits without the use of medications (n = 982), male patients accounted for 60.4% of the visits, and schizophrenic disorders were the most prevalent diagnoses (16.6%). In terms of patients’ diagnosis, 57.2% (n = 3266) of patients with neurotic disorders, 64.0% (n = 1510) of patients with schizophrenic disorders, and 74.2% (n = 2406) patients with affective disorders were managed with three or more medications. A higher proportion of female patients with neurotic disorders (59.8%, n = 1983) than male patients (56.9%, n = 1283) received three or more medications. In patients with schizophrenic disorders and affective disorders, the use of three or more medications were seen in 71.7% of females vs. 70.8% of males and 77.3% of females vs. 76.7% of males, respectively.

As shown in Table 3, the most commonly prescribed drugs were hypnotics and sedatives, followed by antidepressants, anxiolytics, antipsychotics, and antiepileptics. There were more female patients (n = 7029, 58.4%) than male patients (n = 5002, 41.6%) who received hypnotics-sedatives and anxiolytics (n = 12,031). In patients with affective disorders and neurotic disorders, antidepressants were the most common medications, while antipsychotics were the most common medications in patients with schizophrenic disorders.

On the ATC 5th level, zolpidem was the most commonly prescribed psychotropic drug, followed by clonazepam, alprazolam, lorazepam, trazodone, estazolam, and quetiapine (Table 4). Of the patients receiving zolpidem, 62.0% were female (n = 1862). Zolpidem was most commonly prescribed in physician clinics (n = 1320, 44.0%), followed by metropolitan hospitals (n = 810, 27.0%), academic medical centers (n = 466, 15.5%), and local community hospitals (n = 402, 13.4%).

Trazodone was the most popular antidepressant, probably because it was usually used as a hypnotic in Taiwan. Other antidepressants included escitalopram, sertraline, mirtazapine, paroxetine, melitracen, fluoxetine, venlafaxine, citalopram, duloxetine, moclobemide, and milnacipran. Selective serotonin reuptake inhibitors (25.6%) were prescribed more frequently than serotonin norepinephrine reuptake inhibitors (6.3%) and other antidepressants. Among patients receiving antidepressants, 60.0% (n = 3876) were female and 40.0% (n = 2585) were male.

Quetiapine was the most commonly used antipsychotic, followed by risperidone, sulpiride, olanzapine, aripiprazole, haloperidol, clozapine, amisulpride, flupentixol, chlorpromazine, clotiapine, zotepine, paliperidone, trifluoperazine, ziprasidone, thioridazine, zuclopenthixol, and fluphenazine. Second-generation antipsychotics were more commonly prescribed than first-generation antipsychotics (28.3% vs. 11.6%).

Among mood stabilizers, valproic acid was the most commonly prescribed drug, followed by lithium, lamotrigine, and carbamazepine.

Laxatives were the most commonly used non-psychotropic drugs, including senna glycoside, magnesium oxide, and bisacodyl.

## 4. Discussion

In 2012, psychiatric outpatient visits accounted for 2.1% of all ambulatory care visits in Taiwan, which was relatively low compared with 3.1% in the United States [11]. Previous studies showed that the use of outpatient mental health services is associated with the prevalence of mental morbidity and the perceived need for care [12]. The lower proportion of psychiatric outpatient visits among all ambulatory care in Taiwan requires further investigation. The present study found that more outpatient psychiatric visits were made by females than males (54.0% vs. 46.0%). A previous study also demonstrated that females use mental health care services more frequently than males [13], but this pattern depends on the type and severity of mental health problems [14]. Taking only the first diagnosis for each claim into account, there were more female patients than male patients among the patients with neurotic disorders (60.1% vs. 39.9%) and among those with affective disorders (62.7% vs. 37.3%). These findings were consistent with prior epidemiological studies that found a higher risk of mental disorders—especially neurotic disorders and affective disorders [15]—among females than among males, with those types of disorders being the most prevalent diagnoses among psychiatric outpatients. Patients under the age of 60 (73.8%) were the major source of outpatients. This might have been due to the higher prevalence of mental illness among young adults than among older adults [16,17].

In roughly half of outpatient visits (51.7%), hypnotics and sedatives were prescribed. Among those drugs, zolpidem was the most commonly prescribed (22.6%). Compared with male patients, female patients were more commonly prescribed with hypnotics-sedatives and anxiolytics, including zolpidem. Higher rates for the usage of hypnotics among female patients have been demonstrated in previous studies [18], and may be partially explained by higher prevalences of affective disorders, anxiety disorders, and insomnia among female patients [15,19]. In addition, the most common comorbidities associated with insomnia are psychiatric disorders [20,21]. It has been reported that 40% of patients with insomnia have coexisting psychiatric disorders [22]. Depression is the most common comorbidity among these psychiatric disorders [23]. Insomnia comorbid with psychiatric disorders, medical disorders, or substance use accounts for 85%–90% of chronic insomnia [24].

High rates of benzodiazepine and Z-drug usage in Taiwan have been reported in the prior literature. Wang et al. reported an annual prevalence of anxiolytic-hypnotic use of more than 20% in Taiwan. Furthermore, the greatest increases in use between 2002 and 2009 were seen for the drugs clonazepam and zolpidem, which, in our study, were the two most commonly prescribed medications for the psychiatric outpatient population. The annual prevalence of zolpidem use in Taiwan increased from 2.4% in 2002 to 4.2% in 2009 [25]. The relatively higher prevalence of hypnotic use in this study might be explained by the fact that most psychiatric outpatients had psychiatric disorders, including neurotic disorders and affective disorders. Previous studies have shown that long-term use of benzodiazepines is associated with increased risk of adverse effects [26], increased risk of accidents such as falling [27,28], and increased cognitive decline in the elderly [29]. Zolpidem has been considered safer than benzodiazepines, and it is associated with a lower probability of abuse and dependence [30]. However, there is a growing evidence that zolpidem has the potential for abuse and dependence [31,32]. The finding of high prevalence of hypnotics and sedatives use poses significant concerns regarding dependence on these agents and subsequent adverse events. Clinicians should reduce unnecessary use of hypnotics and sedatives and monitor their risk of dependence carefully. Notably, recent studies found that zolpidem is associated with increased risk of motor vehicle collisions [33,34]. Considering the high prevalence of zolpidem use in psychiatric outpatients, clinicians should consider nonpharmacological treatment before initiating zolpidem in this population.

As the most prevalent antidepressant, trazodone was more commonly used as a hypnotic in Taiwan. However, the evidence supporting the use of trazodone as a hypnotic is limited [35,36]. The high prevalence of trazodone use as a hypnotic in Taiwan thus deserves further study to evaluate the underlying causes and potential risks. The trends in the prescription of atypical antipsychotics and the new generation of antidepressants were demonstrated in this study. In 2002, typical antipsychotics were more commonly prescribed than atypical antipsychotics (12% vs. 0.7%) [37], while in 2012, the rate of prescriptions for atypical antipsychotics was more than two times that of typical antipsychotics (27.4% vs. 11.6%). The trend of atypical antipsychotics taking the place of typical antipsychotics has also been reported in other countries [38,39]. Although the atypical antipsychotics have some advantages over typical antipsychotics (such as causing extrapyramidal syndromes less frequently), the increased risk of metabolic effects, including diabetes, hyperlipidemia, and obesity associated with atypical antipsychotics warrants clinical attention [40]. While the rate of prescriptions for non-selective monoamine reuptake inhibitors exceeded that of prescriptions for selective serotonin reuptake inhibitors (3.3% vs. 1.8%) in 2002, selective serotonin reuptake inhibitors (55.1%) were the most prevalent antidepressants as of 2012. A similar phenomenon was also previously documented in the United States [41]. While lithium was the most prevalent mood stabilizer in 2002 (4%), valproic acid had surpassed lithium as of 2012 (6.1% vs. 2.0%) [37]. These changes in mood stabilizer usage rates might be explained by the changing clinical practice in the treatment of bipolar disorders. Trends of increased use of anticonvulsants and decreased use of lithium in the treatment of acute mania in German, Swiss, and Austrian hospitals between 1994 and 2004 were also reported [42]. Although lithium retains a role as a first-line treatment of acute mania, it has narrow therapeutic index and many side effects, including tremor, polydipsia, polyuria, and, in the long term, hypothyroidism [43]. While valproic acid has grown in popularity, prescribers should carefully monitor its side effects, such as teratogenicity, weight gain, hair loss, and hepatotoxicity [44].

The common medical comorbidities among psychiatric outpatients included constipation, hypertension, and diabetes mellitus. It has previously been demonstrated that people with severe mental illnesses have a higher risk of diabetes and hypertension [45,46,47]. In this study, the diagnosis of constipation was found in 5.5% of psychiatric outpatients. The common use of laxatives was reflected by the high prevalence of constipation in our data. Psychotropic drugs and psychiatric comorbidities might be associated with the occurrence of constipation [48,49]. Antipsychotics, tricyclic antidepressants, and selective serotonin reuptake inhibitors have been reported to be associated with increased risk of constipation [48]. 

There were some limits to this study. First, this was a cross-sectional study. The changes and potential trends across time could not be identified due to the lack of longitudinal data. Second, the associations between various mental illnesses and comorbidities were not investigated due to the sampling methods used in this study. Third, the patterns of prescriptions for different diagnoses were not examined. Fourth, data regarding the services provided by general physicians and nonpsychiatrist specialists were not included in this study. Wang et al. previously demonstrated a higher proportion of inadequate psychiatric treatment in general medical sectors than in the mental health specialty [50]. The lack of such data in this study limits the degree to which psychiatric practices for outpatient populations can be assessed accurately. Fifth, prescription information may be under-reported, because the ambulatory data did not include prescription drugs from pharmacies. Sixth, due to the study design, this study focused on the basic features of psychiatric outpatient visits, it being inappropriate to analyze the association between the variables. Finally, the diagnoses listed in the medical claims in the insurance system might not accurately represent patients’ true conditions, and their validity was rarely examined.

## 5. Conclusions

This nationwide population-based study characterized psychiatric outpatient practice in Taiwan. Hypnotics-sedatives and anxiolytics were found to be widely prescribed, especially zolpidem. Further research and policy interventions are necessary to explore the potential abuse of hypnotics-sedatives and anxiolytics in the outpatient population.

## Figures and Tables

**Table 1:** The distribution of diagnoses among psychiatric outpatients with all diagnoses at each claim were taken into account.

ICD9CM	Diagnosis Group	(n = 13,261)	%
300	Neurotic disorders	5714	43.1
296	Affective psychoses	3244	24.5
295	Schizophrenic disorders	2359	17.8
307	Special symptoms or syndromes, not elsewhere classified	2327	17.5
780	General symptoms	1299	9.8
311	Depressive disorder, not elsewhere classified	813	6.1
564	Functional digestive disorders, not elsewhere classified	733	5.5
290	Senile and presenile organic psychotic conditions	693	5.2
314	Hyperkinetic syndrome of childhood	667	5.0
294	Other organic psychotic conditions (chronic)	518	3.9
401	Essential hypertension	516	3.9
309	Adjustment reaction	431	3.3
333	Other extrapyramidal disease and abnormal movement disorders	263	2.0
784	Symptoms involving head and neck	238	1.8
250	Diabetes mellitus	209	1.6

ICD9CM: International Classification of Diseases, 9th Revision, Clinical Modification.

**Table 2:** Numbers and percent distributions of nonpharmacological treatments.

NHI Code	Treatment	(n = 13,261)	%
45010C	Supportive individual psychotherapy	2117	16.0
45087C	Re-educative individual psychotherapy-adult	1578	11.9
45088C	Re-educative individual psychotherapy: 6–15 years old	127	1.0
45100C	Behavior modification assessment	49	0.4
45013C	Intensive individual psychotherapy-adult	34	0.3
45016C	Supportive group psychotherapy	33	0.2
45094C	Re-educative group psychotherapy	24	0.2
45022C	Activity therapy (day)	24	0.2
45043C	Biofeedback therapy	14	0.1
45089C	Re-educative individual psychotherapy-under 6 years old	12	0.1
45095C	Special Occupational therapy	10	0.1
45090C	Intensive individual psychotherapy: 6–15 years old	7	0.1

NHI: national health insurance.

**Table 3:** The prescription of drugs by Anatomical Therapeutic Chemical (ATC) 3rd level.

ATC Code	Drug Classification	n = 13,261	%
N05C	Hypnotics and sedatives	6850	51.7
N06A	Antidepressants	6461	48.7
N05B	Anxiolytics	5181	39.1
N05A	Antipsychotics	4869	36.7
N03A	Antiepileptics	3076	23.2
C07A	Beta blocking agents	1443	10.9
N04A	Anticholinergic agents	1410	10.6
A06A	Laxatives	812	6.1
N06B	Pscyhostimulants	671	5.1
N06C	Psycholeptics and psychoanaleptics in combination	578	4.4

**Table 4:** The prescription of drugs by ATC 5th level.

ATC Code	Drug Classification	n = 13,261	%
N05CF02	Zolpidem	2998	22.6
N03AE01	Clonazepam	2312	17.4
N05BA12	Alprazolam	2222	16.8
N05BA06	Lorazepam	1894	14.3
N06AX05	Trazodone	1675	12.6
N05CD04	Estazolam	1583	11.9
N05AH04	Quetiapine	1352	10.2
C07AA05	Propranolol	1331	10.0
N05CD03	Flunitrazepam	1,102	8.3
N06AB10	Escitalopram	1,089	8.2
